# Background qualitative analysis of the European Reference Life Cycle Database (ELCD) energy datasets – part I: fuel datasets

**DOI:** 10.1186/s40064-015-0915-9

**Published:** 2015-03-28

**Authors:** Daniel Garraín, Simone Fazio, Cristina de la Rúa, Marco Recchioni, Yolanda Lechón, Fabrice Mathieux

**Affiliations:** Energy Department, Energy Systems Analysis Unit, CIEMAT, Av. Complutense 40, E28040 Madrid, Spain; European Commission, Joint Research Centre, Institute for Environment and Sustainability, Unit JRC.H.8-Sustainability Assessment, Via E. Fermi 2749 - TP 270, 21027 Ispra, Italy

**Keywords:** ELCD database, Energy datasets, Fuel, Biofuel, Natural gas, Data quality indicators

## Abstract

**Introduction:**

The aim of this study is to identify areas of potential improvement of the European Reference Life Cycle Database (ELCD) fuel datasets.

**Case description:**

The revision is based on the data quality indicators described by the ILCD Handbook, applied on sectorial basis. These indicators evaluate the technological, geographical and time-related representativeness of the dataset and the appropriateness in terms of completeness, precision and methodology.

**Discussion and evaluation:**

Results show that ELCD fuel datasets have a very good quality in general terms, nevertheless some findings and recommendations in order to improve the quality of Life-Cycle Inventories have been derived. Moreover, these results ensure the quality of the fuel-related datasets to any LCA practitioner, and provide insights related to the limitations and assumptions underlying in the datasets modelling.

**Conclusions:**

Giving this information, the LCA practitioner will be able to decide whether the use of the ELCD fuel datasets is appropriate based on the goal and scope of the analysis to be conducted. The methodological approach would be also useful for dataset developers and reviewers, in order to improve the overall DQR of databases.

## Introduction

The European Platform of Life Cycle Assessment (EPLCA), a project initiated by the Institute for Environment and Sustainability (IES), has the objective of promoting Life Cycle Thinking (LCT) and providing appropriate support to business and public administrations within the European Union (EU), as well as in close coordination with international activities. This support is essential, and is being achieved through the development of a number of different deliverables, being the European Reference Life Cycle Database (ELCD) one of them (Recchioni et al. 2014). The ELCD provides core Life Cycle Inventory (LCI) data from front-running EU-level business associations and, where not available, other sources. Several energy-related datasets are provided within the ELCD, since energy is a key input to most environmental analyses of products or processes. The ELCD latest version can be consulted on the JRC webpage: http://eplca.jrc.ec.europa.eu/ELCD3/.

Although LCA-based methodologies and tools seem to develop fast, the availability of quality-assured LCA data still represents a major bottleneck to a broader use of LCA and environmental footprint methods in business and in policy (Fazio et al. 2015). Under the framework of ISO standards (ISO 2006) some guidelines have been developed to address the Data Quality Requirements: i) ILCD handbook (EC-JRC 2010a) considers six indicators regarding technological representativeness, geographical representativeness, time-related representativeness, completeness, precision/uncertainty, and methodological appropriateness and consistency, ii) UNEP/SETAC life cycle initiative (UNEP 2011) also include reproducibility, representativeness, and information on data sources, iii) USLCI Database (2012) Project Development Guidelines describes the data quality basing on data age, source and collection method; data representativeness; averaging methods; methods used to estimate or justify data gaps; and information about key assumptions or methodological choices, and iv) Ecoinvent (http://ecoinvent.ch) proposes a specific Data Quality rating, named pedigree matrix, where include aspects such as geographical, technological and temporal validity, the origin, representativeness and validation of the data, and administrative information (Fazio et al. 2015).

The objective of this analysis is to identify areas of potential improvement of the ELCD fuel datasets quality, considering data available in third party life cycle databases and from authoritative bodies and/or business associations. The work has consisted in analysing and comparing fuel datasets from different databases, considering the ELCD database as the basis for this analysis. This effort has been carried out in two stages, which are summarized below:

- Selection of datasets, databases and quality standards, in order to assure the methodology. This part aimed at providing a justified list of datasets and databases (and other sources) to consider in the subsequent analysis. Moreover, justified criteria and quality standards list have been clearly defined in order to be used in the analytical comparison.
- Analysis and qualitative comparison of the datasets. Each selected fuel dataset was analysed according to the previously defined quality indicators. Then, findings and recommendations were derived in order to identify the potential improvements of ELCD datasets.

## Methods

### Selection of datasets and databases

The energy datasets to be analysed should be representative of the European context, and therefore a deep review of the most updated data in terms of fuel for EU -27 has been conducted.

According to European statistics (EUROSTAT 2012; EC 2011), there are four main petroleum products obtained from the European refineries: i) Diesel, which represents more than 37% of the refineries output; ii) Gasoline, which represents more than 20%; iii) Residual (or Heavy) fuel oil, which represents more than 15%; and iv) Kerosene, that represents more than 6%. Due to their relevance in the share of fuel production, these products were chosen for the analysis to be conducted.

Biofuels production has significantly increased during the last decade due to a favourable framework and the support of several policies. Nowadays biofuels represent 11% of the total biomass produced in EU-27, being biodiesel the highest contributor to the total production, 60% (EC 2011). The contribution of Europe to biofuels production is expected to increase due to its high potential. Nevertheless, this affirmation could be misleading because of two reasons: i) According to many studies the potential for European biofuels production is clearly limited, and ii) Currently (2013), a substantial share of biofuels used in Europe is based on imported feedstock. In case of increasing, rapeseed oil seems to be one of the raw materials expected to contribute the most in the share of biodiesel. So, in order to cover this potential fuel in the analysis, biodiesel from rapeseed oil was included as dataset.

Additionally, an analysis of the gross heat generation in the EU-27 pointed out the relevance of the natural gas as fuel, being its contribution to the heat generation around 44% (EC 2011). Then, natural gas was considered as the most important heat supplier dataset in the analysis.

The current fuel datasets available at the ELCD database have been originated from PE International (GaBi developers). The latest ELCD includes fuel datasets referring to EU-15. Since the scope of this evaluation is to analyse the ELCD datasets under the European context, it seems appropriate the use of datasets from GaBi that consider EU-27 as geographical horizon. Table 1 shows the six chosen datasets as the base for the comparison with other datasets.

**Table 1:** **List of the selected ELCD fuel datasets as basis for comparison**

**Fuel**	**Location**	**Name of LCI process/dataset**
Crude oil and natural gas based fuels	EU-27	Diesel mix at refinery
	EU-27	Gasoline mix (regular) at refinery
	EU-27	Heavy fuel oil at refinery (1.0 wt.% S)
	EU-27	Kerosene/Jet A1 at refinery
	EU-27	Natural gas mix
Biofuel (Rapeseed ,methyl ester, RME)	Germany	DE: Rapeseed methyl ester (RME)

These datasets have been compared to their counterparts from three other databases, which have been selected based on three main criteria: i) they include data related to Europe, ii) they include large data related to energy products and services, and iii) they are well recognised in the scientific community. The selected databases have been the following: Ecoinvent v2.2, GEMIS 4.7, and E3 database. Considering theses databases and the availability of datasets, Table 2 presents the list of datasets to be finally analysed. The database selection have been made irrespective of the methodological compliance of the database/datasets with the ILCD quality criteria: it was indeed assumed that although other databases might have lower DQR according to ILCD rules (because they were not specifically developed using these rules), datasets would represent interesting benchmarks and some improvement could be derived from the background analysis (Fazio et al. 2015).

**Table 2:** **Selected datasets to be analysed by database**

**ELCD**	**Ecoinvent v2.2**	**GEMIS 4.7**	**E3**
EU-27: Diesel mix at refinery	Diesel, at refinery/RER	Refinery\Diesel-generic	Diesel-2010/Crude oil refinery
EU-27: Gasoline mix (regular) at refinery	Petrol, low-sulphur, at refinery/RER	Refinery\Gasoline-generic	Gasoline-2010/Crude oil refinery
EU-27: Heavy fuel oil at refinery (1.0 wt.% S)	Heavy fuel oil, at refinery/RER	Refinery\Oil products-generic	Fuel oil/Heavy/Provision
EU-27: Kerosene/Jet A1 at refinery	Kerosene, at refinery, RER	Refinery\Kerosene (int)	-
EU-27: Natural gas mix	Natural gas, at long distance pipeline, RER	Gas-mix-EU 2005	NG/Extraction + processing
DE: Rapeseed methyl ester (RME)	Rape methyl ester, at esterification plant/RER	Refinery\Rapeseed oil-ME-iLUC(50%) (arable)	FAME/Plant oil/Esterification

### Quality criteria for analysis

The evaluation has been based on the quality indicators developed within the ILCD handbook (EC-JRC 2010a, b, 2011): Technological representativeness (TeR), Geographical representativeness (GR), Time-related representativeness (TiR), Completeness (C), Precision/Uncertainty (P) and Methodological appropriateness and consistency (M). Each of those has been evaluated according to the degree of accomplishment of the criterion, from 1 (very good, so meets the criterion to a very high degree) to 5 (very poor, so does not at all meet the criterion).

An overall Data Quality Rating (DQR) of the datasets has been calculated by summing up the achieved quality rating for each of the quality criteria indicator, divided by the total number of considered indicators, as shown in Equation 1.

1 $$ DQR=\frac{TeR+GR+TiR+C+P+M}{6} $$

According to ILCD Handbook (EC-JRC 2011), an overall data quality level can be defined regarding the DQR value, as shown in Table 3.

**Table 3:** **Overall data quality level according to DQR (EC-JRC** 2011 **)**

**DQR**	**Overall data quality level**
≤ 1.6	Excellent quality
> 1.6 to ≤ 2.0	Very good quality
> 2.0 to ≤ 3.0	Good quality
> 3.0 to ≤ 4.0	Fair quality
> 4.0	Poor quality

Nevertheless, it should be noticed that a single score indicator might lead to misleading interpretation of the results. Some datasets might not contain enough information to evaluate them against all criteria and summing all scores could be misunderstood. The analysis is focused on the improvement of ELCD fuel datasets and has been based on the available documentation and/or information of database providers. The unavailability of certain information does not automatically mean that a dataset is potentially worse than another.

The quality indicators described in the ILCD Handbook (EC-JRC 2011) provide a general framework to evaluate datasets. When applying these indicators to specific sectorial datasets, it is necessary to redefine them based on the specific characteristics of the processes/technologies in order to identify key aspects. This practice facilitates their use in the analysis of fuel energy systems. For this purpose, a deep pre-analysis of the technology situation was conducted, considering the European market context. The main features for assessing each criterion are summarized below (Fazio et al. 2015). Table 4 highlights both quality criteria definitions and values considered.

**Table 4:** **Matrix for assessing LCI of fuel datasets**

**Quality indicator**	**Subquality parameters**	**Rating**				
		**1 (Very Good)**	**2 (Good)**	**3 (Fair)**	**4 (Poor)**	**5 (Very Poor)**
**TeR**	Expert judgement based on the consideration of a technology mix	Technology aspects have been modelled as the technology mix	Technology aspects are very similar to the technology mix	Technology aspects are similar to the technology mix	Technology aspects are different to the technology mix	Technology aspects are completely different to the technology mix, or tech not deployed
**GR**	Expert judgement based on the geographical coverage of data	Involved countries fulfil completely the share of listed as referenced countries	Involved countries fulfil very similarly the share of listed as referenced countries	Involved countries fulfil similarly the share of listed as referenced countries	Involved countries fulfil differently the share of listed as referenced countries	Involved countries fulfil completely different the share of listed as referenced countries
**TiR**	Expert judgement based on defined time on data inventory (± 5 years)	All the data sources refer to the defined time	The majority of the data sources refer to the defined time	At least half of the data sources refer to the defined time	Less than half of the data sources refer to the defined time	None the data sources refer to the defined time
**C**	Consideration of impact categories and share of elementary flows (to adjust the final rating)	15-16 considered impact categories	12-14 considered impact categories	8-11 considered impact categories	5-7 considered impact categories	≤5 considered impact categories
**P**	Expert judgement based on the precision/uncertainty of data sources	Very low uncertainty and/or very high precision	Low uncertainty and/or high precision	Fair uncertainty and/or fair precision	High uncertainty and/or low precision	Very high uncertainty and/or very low precision
**M**	Definition of situation context and subsequent expert judgement of system boundaries, multi-functionality and EoL	Inclusion of all LCA stages (with the EoL stage). Consideration of allocation procedures. Completion in a very high degree	Inclusion of most relevant LCA stages. Consideration of allocation procedures. Completion in a high degree	Inclusion of a still sufficient LCA stages. Consideration of allocation procedures. Completion in a sufficient degree	Inclusion of a sufficient LCA stages. Consideration of allocation procedures. Completion in a low degree	No inclusion of sufficient LCA stages. No consideration of allocation procedures (multi-functionality has not been solved according to the situation context). Completion in a low degree

- **TeR**, **GR** and **TiR** representativeness: These criteria define the degree to which datasets reflect true population of interest regarding technology, geography and time/age of the data, respectively. Datasets related to the most representative fuel technologies in each area, in the European market context, basing on the above mentioned statistic criteria derived from authoritative sources. The origins of the imported raw fuels (if any) for fuel production, have been listed for each chosen country. TiR has been related to the expected obsolescence of the technology applied (based on existing data) defined as the year/s in which inventory was collected, with a deviation of ± 5 years. The framework is the same proposed by the ILCD Handbook, however the sector-specific expert judgement has been used to define the above mentioned criteria (e.g. the adjustment on elementary flows coverage, quality of references, etc.), through the analysis of authoritative sources.
- **C**: Defines the share of (elementary) flows that are quantitatively included in the inventory and should assess the degree of coverage of the overall environmental impact. It is assessed as the share of elementary flows, weighted on the number of environmental impact categories that are quantitatively included in the inventory. A pre-analysis based on sectorial experience, to identify the elementary flows (and within them a list of the most relevant ones in mass and/or impact basis) that allow the estimation of the 16 environmental impact categories mentioned at the mid-point level ILCD 2011 recommended method was done – see EC (2011) -. In this paper the preliminary analysis on completeness lead to a score based on the number of covered impact categories, then the score have been adjusted in relation to the coverage of relevant elementary flows included in the dataset (i.e. no changes if the flow list includes more than 75% of the flows, one level lower if the flow list is covered only from 50 to 75%, and 2 levels lower if the flow coverage is less than 50%)
- **P:** Defines the measure of the variability of the data values for each data expressed. Decisive factors accounted were both the reliability of data and the uncertainty degree of the information (such as data, models and assumptions). Thus an expert judgement has been considered, based on the quality of the references and their sources, whether measured, calculated or estimated from literature.
- **M:** Defines if the applied LCI methods and methodological choices are in line with the goal and scope of the data set, especially its intended applications and decision support context. To evaluate this criterion, a correct and consistent application of the recommended LCI modelling framework and LCI method approaches for the given situation, according to the ILCD Handbook, was applied, focusing on three issues: i) System boundaries; ii) End of Life (EoL) modelling iii) Multifunctionality (according to the different contexts defined in the ILCD Handbook.

## Results

Table 5 shows the rates of the quality criteria assessment of the selected ELCD fuel datasets. Information contained in the dataset and additional confidential documents provided by the database developer (PE 2012a) were considered to define a final single value for each criterion.

**Table 5:** **Quality criteria and DQR values of fuel ELCD datasets**

**Datasets**	**Database**	**DQI**	**Score**	**Short justification of DQI**	**DQR**
Diesel mix, Gasoline mix and Heavy fuel oil (1.0 wt.% S) at refinery	ELCD	TeR	1	Relevant primary and secondary data referred to EU27	1.08
		GR	1	Very good modelling of EU27 share and market relevance	
		TiR	1	Ref year 2009, data from 2007 to 2009	
		C	1	16 (100%) impact categories, 96% of flows covered	
		P	1-2	Some data are calculated basing on technical descriptions	
		M	1	Cradle to grave process, EoL and infrastructure included	
	Ecoinvent	TeR	2	Some transport distances refers to Swiss refineries	1.75
		GR	2	Few countries not included	
		TiR	1-2	Ref year 2000, some data from ‘80s	
		C	1	16 (100%) impact categories and 100% of reference flows covered	
		P	2	Some oil extraction data from Africa are roughly estimated	
		M	2	EoL not modelled, infrastructure and allocation included	
	GEMIS	TeR	3	Modelled by a generic plant, default distance values	3.50
		GR	5	Not referred to any specific country	
		TiR	4	Ref year 2000, data from 1985 to 95	
		C	2	75% of impact categories, 90% of flows covered	
		P	4	Estimated data from literature, assumptions not disclosed	
		M	3	EoL not comprised, Allocation not specified	
	E3	TeR	2	Modelled from JEC (2007) report assuming oil from middle east	2.67
		GR	3	Extraction only from mid. east, representativeness of EU refinery system is not explained	
		TiR	2	Ref. year 2010, data coming from JEC (2007): 1996-2007	
		C	4	Less than 50% of impact categories, 90% of flows covered	
		P	2	No info about emission factors	
		M	3	Cradle to gate system, EoL not included.	
Kerosene/Jet A1 at refinery	ELCD	TeR	1	Relevant primary and secondary data referred to EU27	1.08
		GR	1	Very good modelling of EU27 share and market relevance	
		TiR	1	Ref year 2009, data from 2007 to 2009	
		C	1	16 (100%) impact categories, 96% of flows covered	
		P	1-2	Some data are calculated basing on technical descriptions	
		M	1	Cradle to grave process, EoL and infrastructure included	
	Ecoinvent	TeR	2	Some transport distances refers to Swiss refineries	1.75
		GR	2	Few countries not included	
		TiR	1-2	Ref year 2000, some data from ‘80s	
		C	1	16 (100%) impact categories and 100% of reference flows covered	
		P	2	Some oil extraction data from Africa are roughly estimated	
		M	2	EoL not modelled, infrastructure and allocation included	
	GEMIS	TeR	4	Modelled by a generic plant	3.83
		GR	5	Referred to an Indian refinery	
		TiR	4	Ref year 2000, data from 1990 to 1996	
		C	2	75% of impact categories, 90% of flows covered	
		P	4	Estimated data from literature, assumptions not disclosed	
		M	4	EoL not comprised. Allocation applied but not defined	
Natural gas mix	ELCD	TeR	1	Relevant indigenous and import NG data referred to EU27. Supply is included	1.00
		GR	1	Very good modelling of EU27 share and market relevance	
		TiR	1	Ref year 2009, data from 2007 to 2009	
		C	1	16 (100%) of impact categories, 98% of flows covered	
		P	1	European and World Statistics as sources	
		M	1	Cradle to grave process, EoL and infrastructure included	
	Ecoinvent	TeR	1	Modelled regarding a European standard mix. Average distances	1.67
		GR	2	Few countries not included	
		TiR	2	Ref year 2000, very few data does not cover time horizon	
		C	1	16 (100%) impact categories and 100% of reference flows covered	
		P	2	NG production data from environmental reports. Average data used for some countries	
		M	2	EoL not modelled, infrastructure and allocation included	
	GEMIS	TeR	3	NG exploration focused on several countries. Distances as typical value	2.83
		GR	3	Minor countries are not considered. EU-25 is considered in 2005	
		TiR	2	Ref year 2005, data from 1990 to 2006	
		C	2	75% of impact categories, 90% of flows covered	
		P	4	Estimated data from literature, assumptions not disclosed	
		M	3	EoL not comprised. Allocation applied but not defined. Infrastructure is included. Not possible to identify different stages.	
	E3	TeR	3	Modelled from JEC (2007) report (EU mix). Supply from personal communications. Average distances.	3.58
		GR	3	EU-27 is considered but not possible to identify each country share	
		TiR	3	Ref. year 2006, data coming from ‘90s	
		C	4	Less than 50% of impact categories, 90% of flows covered	
		P	4	No info about emission factors, hypotheses and assumptions	
		M	4-5	Cradle to gate system, EoL not included, allocation not defined	
DE: Rapeseed methyl ester (RME)	ELCD	TeR	2	Consideration of the whole process, except rapeseed and oil imports	2.20
		GR	3	Modelling as region specific in Germany	
		TiR	2	Ref year 2010, data from 1996 to 2001	
		C	1	15 impact categories, 93% of flows covered	
		P	n/a	Flows come from literature, but no enough info for many processes	
		M	3	Cradle to grave process, EoL and infrastructure are not included	
	Ecoinvent	TeR	2	Swiss transesterification plant in EU conditions. Not imported rapeseed or oil is considered	1.67
		GR	3	German condition of farming. No imports are considered	
		TiR	1	Ref year 1996-2000, data from 1996 to 2010	
		C	1	16 (100%) impact categories and 100% of reference flows covered	
		P	1	Literature review, official sources of data and some primary data	
		M	2	EoL not modelled, infrastructure and market allocation included	
	GEMIS	TeR	2	German conditions of transesterification. No info about type of plants and/or equipment. Not imports are included	2.33
		GR	3	German condition of farming. No imports are considered	
		TiR	2	Ref year 2010, data from 1999 to 2010	
		C	2	75% of impact categories, 90% of flows covered	
		P	3	Data come from literature review	
		M	2	EoL not comprised. Heat value allocation applied. Infrastructure is included. ILUC considered	
	E3	TeR	2	Production of rapeseed, oil and RME in EU are included, but no imports	3.00
		GR	3	European conditions	
		TiR	3	Ref year 2010, data from 1995 to 2002	
		C	4	Less than 50% of impact categories, 90% of flows covered	
		P	3	Data come from literature review	
		M	3	Cradle to gate system, EoL not included, energy allocation included	

*n/a: not assessed due to lack of data.

## Discussion

The comparison of the selected datasets from different databases, referred to the same technology, can lead to the identification of potential improvements in each quality criteria. Moreover, relevant Authoritative Sources and Business Associations, which could provide additional information to improve the quality of the ELCD results, can be also identified in order to enhance the overall quality of data. It must be remarked that many recommendations are related to future updated versions of ELCD fuel datasets. Table 6 shows a summary of the findings and recommendations that arose from such cross assessment.

**Table 6:** **Recommendations for improving ELCD fuel datasets by DQI**

**ELCD datasets**	**DQI**	**Potential improvements and recommendations**
Diesel mix, Gasoline mix, Heavy fuel oil (1.0 wt.% S), and kerosene/jet A1 at refinery	TeR and P	• Score could improve by using the most updated version of the JEC (2011). However, it is necessary to highlight that the JEC project is not an LCA study, as the study recognizes itself, but a well to wheel study limited to energy and greenhouse gas emissions. Furthermore, since it focuses on future powertrains, some assumptions do not truly reflect current practices.
	C	• In order to meet the criterion in a 100% share the following flows have to be considered: CFC-11 and CFC-12 for ozone depletion; and Decane for freshwater ecotoxicity.
	M	• Allocation in ELCD datasets has been performed applying the so-called ‘Back-Pack principle’ methodology (PE 2012b). This is a non-usual allocation procedure to assign a ‘backpack’ of allocated crude oil, energy and electricity demand to each output of the refinery unit processes. This practice partially accomplishes the subdivision procedure highly recommended by ILCD Handbook (EC-JRC-IES 2010a), avoiding black box unit scenarios. The handbook suggests a partially/virtually subdivision of process chains to collect data exclusively for those included processes that have only the required functional outputs.
	General	• ELCD takes advantages of the well-recognized E-PRTR (http://prtr.ec.europa.eu), which produces key environmental data from industrial facilities in European Union Member States and in Iceland, Liechtenstein, Norway, Serbia and Switzerland.
Natural gas mix	TeR and TiR	• Eurostat should be also reviewed, as an Authoritative Source, for updating future versions. The natural gas mix in Europe in 2011 can be consulted on the web-site (EUROSTAT 2012).
		• Other Business Associations, like Eurogas (European Association of Gas Wholesale, Retail and Distribution Sectors, www.eurogas.be) publishes public EU data facts and statistics of natural gas production and distribution that can be useful for achieving a more updated inventory.
		• Other Authoritative Source that could be useful in future version is the Gas Infrastructure Europe (www.gie.eu.com), a European association representing the infrastructure industry of natural gas, such as the Transmission System Operators, Storage Systems Operator and Terminal Operators. Technical data can be also reviewed from the Technical Association of the European Natural Gas Industry MARCOGAZ (www.marcogaz.org).
		• Unconventional hydrocarbons exploitation such shale gas is a hot topic currently in Europe. Several Member States of the EU are discussing new regulations to allow the exploitation of these resources. Under this framework, the EC is already studying the potential environmental impacts and health risks that may arise from individual projects and cumulative developments of this technology. Taken into account this context, it is recommended to follow the development of this technology and the regulatory framework, so that the technology could be included in future versions, if necessary.
	C	• In order to achieve the criterion in a 100% share, CFC-11 and CFC-12 for ozone depletion impact category have to be considered
	P	• Providing documentation related to the data collection process and additional references to identify the origin of the data values could be useful to achieve a better rating. Although some references provided in the dataset are labeled as Authoritative Sources or Business Associations, it has not been possible to find them.
	General	• It has been modelled in a way that includes the most updated and precise natural gas supply mix in EU-27.
Rapeseed methyl ester (DE)	TeR and GR	• Dataset lacks the consideration of raw material imports –rapeseed and rapeseed oil- . Important differences can appear especially in the cropping systems of rapeseed in exporter countries such as Australia, Ukraine and Russia. Considering these systems would improve the TeR of the rapeseed biodiesel produced in Europe. GR criterion also scores lower due to the same reason.
	TiR	• Many of the references do not cover the reference period. The Ecoinvent dataset performs better in this criterion since its validity year is closer to the years of the references but not due to the use of more recent references.
	C	• In order to achieve the criterion in a 100%, the following flows should be considered: Halon 1211 and CFC-10 for ozone depletion; and iridium, cadmium and cypermethrin for resource depletion.
	M	• ELCD dataset is modelled following a methodological approach that shows important discrepancies with the proposal from the EU Directive 28/2009 (RED 2009). Most important differences are related to allocation procedures of co-products and electricity produced in CHP. Based on this, it would be advisable to harmonize the methodology used in the ELCD database with the methodology proposed by the EC in the framework of biofuels sustainability certification. In order to do that, the E3 dataset can be taken as a reference.
	General	• European Commission Energy Transparency Platform (http://ec.europa.eu/energy/renewables/transparency_platform/transparency_platform_en.htm) could be a source of relevant information.

## Conclusions and recommendations

This extended analysis of the ELCD fuel datasets aimed at providing better founded information related to its data quality, following the indicators developed and described within the ILCD handbook (EC-JRC 2011). This analysis, together with the ELCD electricity datasets one (Garraín et al. 2015), have meant an opportunity to implement these quality indicators to different datasets for the first time. It has had two main consequences. Firstly, the implementation of the quality indicators to the energy-related datasets from the ELCD has been used to understand the room for improvement in future ELCD versions. Additionally, it has also served to identify whether these data quality indicators are applicable and useful for database developers in general, as well as for LCA practitioners. It should be stated that results obtained from this analysis ensure the quality of the energy-related datasets to any LCA practitioner, and provide insights related to the limitations and assumptions underlying in the datasets modelling. Giving this information, the LCA practitioner will be able to decide whether the use of the ELCD datasets is appropriate based on the goal and scope of the analysis to be conducted.

Along the current analysis, several assumptions have been made in order to facilitate the analysis, such as the selection of databases and datasets or the definition of DQIs. The results have to be understood under this context. Taking those considerations into account, the data quality assessment conducted in here should not be extrapolated to datasets under different contexts. Furthermore, the analysis has been performed only to the most representative fuel datasets from the ELCD as well as from the selected databases. The conclusions obtained in this analysis cannot be extrapolated to other type of datasets, nor can be used to compare databases among them.

From the deep analysis conducted, it must be highlighted that the ELCD datasets have been modelled based on an extensive review of the most relevant literature and statistics. The documentation used to model the ELCD energy related datasets can be found in the Life Cycle Thinking Platform web-site (http://eplca.jrc.ec.europa.eu/ELCD3/).

In terms of the quality criteria, the analysed ELCD datasets showed a very good performance in the majority of the criteria, where several recommendations for improving have been detailed above. In the case of biofuels and although it fully complies with the methodology quality criterion, it would be advisable to harmonize the methodology recommended by the ILCD handbook and used in the biofuels ELCD datasets with the proposed by the EC in the framework of biofuels sustainability certification. E3 database fully follows this methodology and can be used as a source of data. The EC Energy Transparency Platform is also a source of relevant information.

Concerning the different technologies analysed, crude oil fuel based ELCD datasets achieve the best scores in all quality criteria. It is acknowledged the extensive use of Authoritative Sources and Business Associations as a source of data and the effort to apply an innovative allocation methodology avoiding black box unit scenarios. Natural fuel ELCD dataset performs better than any other database in five quality criteria. It has been modelled in a way that includes the most updated and precise natural gas supply mix in EU-27. The rapeseed biodiesel ELCD dataset has been analysed using the information provided by PE (2012a). Some information was missing and therefore, it could not be evaluated. The dataset lacks the raw material imports –rapeseed and rapeseed oil- which is considered to be a big limitation that should be improved. Regarding the methodology, as mentioned before, there is a lack of harmonization between the methodology used in the ELCD database and the methodology proposed by the EC in the framework of biofuels sustainability verification.

Considering the new research lines of the EC and the social debate generated from the potential shale gas exploitation in Europe, it is recommended to follow the development of this technology and the regulatory framework, so that the technology could be included in future versions, if necessary.

Regarding the use of authoritative sources, the ELCD database makes extensive use of the statistical information provided by the IEA (International Energy Agency). Although the IEA is of course an important authoritative source, for the European context it seems appropriates the use of data reported by each country to Eurostat. In order to improve precision, it would be advisable to make a more extensive use of Business Associations and Authoritative sources data that have been proposed through the analysis.

This analysis aims at providing guidance for the improvement of the fuel ELCD datasets in future versions. Since its first release, the ELCD database has been updated two times. The needs of reviewing and updating the ELCD database depend on the different sectors and the technologies. It would be useful to define periods to revise the fuel related datasets. For this purpose, a deep analysis of the learning curves would identify the level of maturity for new technologies (2^nd^ and 3^rd^ generation biofuels, fuel cells, etc.).

Finally, it should be noted that the selected databases are in a constant process of updating and improvement, *e.g.* Ecoinvent v3.0 or GEMIS v4.93, so a detailed analysis of these can offer further potential improvements to future ELCD versions.

## References

[CR1] EC-JRC (2010). International Reference Life Cycle Data System (ILCD) Handbook – General guide for Life Cycle Assessment – Detailed guidance, European Commission - Joint Research Centre - Institute for Environment and Sustainability, First edition March 2010. EUR 24708 EN.

[CR2] EC-JRC (2010). International Reference Life Cycle Data System (ILCD) Handbook - Specific guide for Life Cycle Inventory data sets, European Commission - Joint Research Centre - Institute for Environment and Sustainability, First edition March 2010. EUR 24709 EN.

[CR3] EC-JRC (2011) International Reference Life Cycle Data System (ILCD) Handbook- Recommendations for Life Cycle Impact Assessment in the European context, European Commission-Joint Research Centre - Institute for Environment and Sustainability, First edition November 2011. EUR 24571 EN. Publications Office of the European Union, Luxemburg

[CR4] EUROSTAT (2012) http://www.eurostat.eu. Access March 2012

[CR5] Fazio S, Garraín D, Mathieux F, de la Rúa C, Recchioni M, Lechón Y (2015) Method applied to the background analysis of energy data to be considered for the European Reference Life Cycle Database (ELCD). Springer Open (accepted, 2015) 10.1186/s40064-015-0914-x PMC4395625 25897408

[CR6] Garraín D, Fazio S, de la Rúa C, Recchioni M, Lechón Y (2015). Mathieux F (2015) Background qualitative analysis of the European Reference Life Cycle Database (ELCD) energy datasets – Part II: Electricity datasets. Springer Open.

[CR7] ISO (2006). ISO 14044:2006 Environmental management - Life cycle assessment - Requirements and guidelines.

[CR8] JEC (2007). JRC-EUCAR-CONCAWE collaboration, Well-to-wheels Analysis of Future Automotive Fuels and Powertrains in the European Context, WELL-to-WHEELS Report, Version 2c, March.

[CR9] JEC (2011). JRC-EUCAR-CONCAWE collaboration, Well-to-wheels Analysis of Future Automotive Fuels and Powertrains in the European Context, WELL-to-WHEELS Report, Version 3c, July.

[CR10] PE (2012). Documentation of energy datasets in GaBi 5.0. Confidential report provided by PE Inernational, Sttutgart, Germany.

[CR11] PE (2012). The GaBi refinery model.

[CR12] Recchioni M, Mathieux F, Blengini GA (2014). Challenges and opportunities for wide publication of quality-assured LCI data: the experience of the Life Cycle Data Network. Int J Life Cycle Ass.

[CR13] RED (2009) Directive 2009/28/EC of the European Parliament and of the Council of 23 April 2009 on the promotion of the use of energy from renewable sources and amending and subsequently repealing Directives 2001/77/EC and 2003/30/EC. Official Journal of the European Union, 5th June

[CR14] UNEP-SETAC (2011). Global guidance principles for life cycle assessment databases. A basis for greener processes and products.

[CR15] USLCI Database (2012). National renewable energy laboratory.

